# Supplementary material for: Stability-mediated epistasis constrains the evolution of an influenza protein
Source: eLife. 2013 May 14;2:e00631. doi: 10.7554/eLife.00631 (PMC3654441; doi:10.7554/eLife.00631)
Supplement: Figure 2—source code 1. — This code is in the form of a ZIP file with BEAST input files and Python code. A README file explains the contents. DOI: http://dx.doi.org/10.7554/eLife.00631.005 [file elife00631s001.zip › elife00631s001/elife00631s001.zip!/Figure_2-source_code_1/mutationdates.pdf]

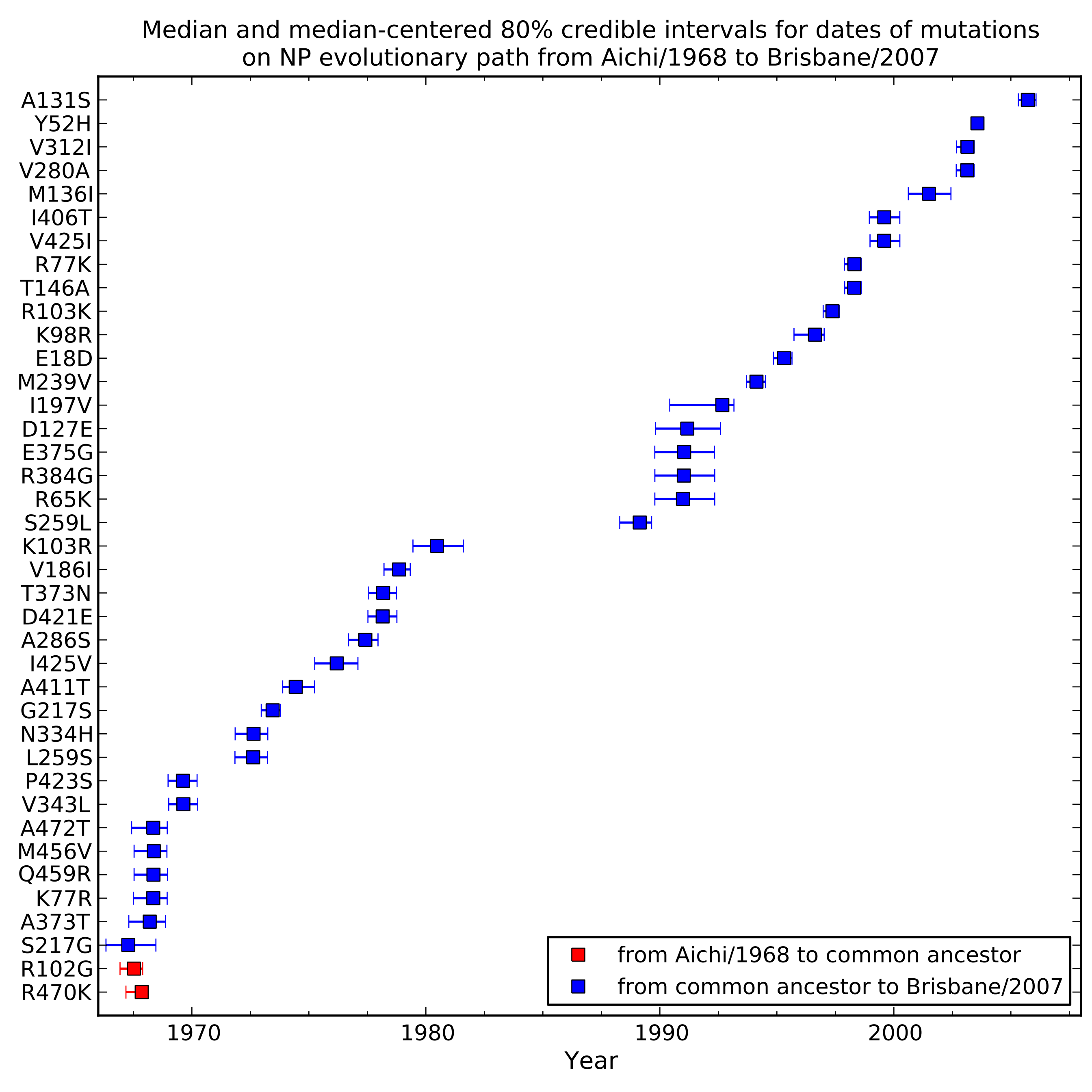
Median and median-centered 80% credible intervals for dates of mutations
on NP evolutionary path from Aichi/1968 to Brisbane/2007
A131S
Y52H
V312I
V280A
M136I
I406T
V425I
R77K
T146A
R103K
K98R
E18D
M239V
I197V
D127E
E375G
R384G
R65K
S259L
K103R
V186I
T373N
D421E
A286S
I425V
A411T
G217S
N334H
L259S
P423S
V343L
A472T
M456V
Q459R
K77R
A373T
S217G
R102G
R470K
from Aichi/1968 to common ancestor
from common ancestor to Brisbane/2007
1970
1980
1990
2000
Year
