# Supplementary material for: Stability-mediated epistasis constrains the evolution of an influenza protein
Source: eLife. 2013 May 14;2:e00631. doi: 10.7554/eLife.00631 (PMC3654441; doi:10.7554/eLife.00631)
Supplement: Figure 8—source code 1. — The code and data are provided as a ZIP file, which contains a README text file that describes the contents in more detail. DOI: http://dx.doi.org/10.7554/eLife.00631.033 [file elife00631s008.zip › elife00631s008/elife00631s008.zip!/Figure_8-source_code_1/plots/epitopedensity_predicted_384-259-280.pdf]

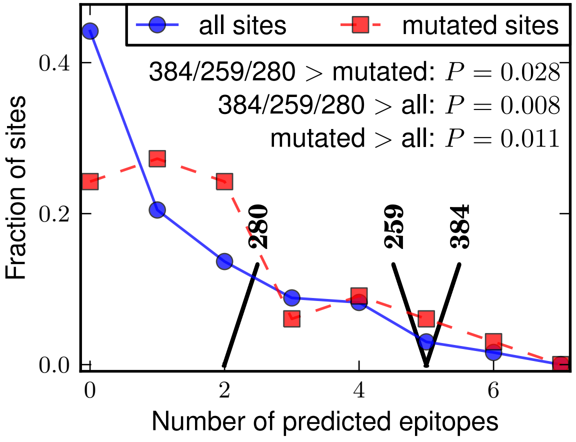

all sites
mutated sites
384/259/280 > mutated: $P = 0.028$
384/259/280 > all: $P = 0.008$
mutated > all: $P = 0.011$
280
259
384
0.0
0.2
0.4
0
2
4
6
Fraction of sites
Number of predicted epitopes
