# Supplementary material for: Stability-mediated epistasis constrains the evolution of an influenza protein
Source: eLife. 2013 May 14;2:e00631. doi: 10.7554/eLife.00631 (PMC3654441; doi:10.7554/eLife.00631)
Supplement: Figure 8—source code 1. — The code and data are provided as a ZIP file, which contains a README text file that describes the contents in more detail. DOI: http://dx.doi.org/10.7554/eLife.00631.033 [file elife00631s008.zip › elife00631s008/elife00631s008.zip!/Figure_8-source_code_1/plots/mapped_epitopes.pdf]

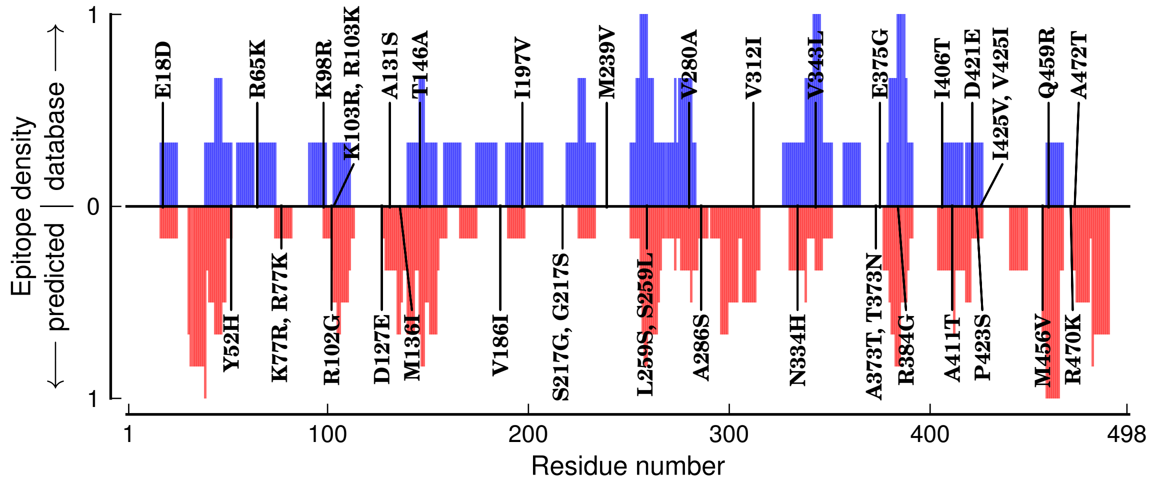
Epitope density
predicted | database
1
0
1
1
100
200
300
400
498
Residue number
E18D
R65K
K98R
K103R, R103K
A131S
T146A
I197V
M239V
V280A
V312I
V343L
E375G
I406T
D421E
I425V, V425I
Q459R
A472T
Y52H
K77R, R77K
R102G
D127E
M136I
V186I
S217G, G217S
L259S, S259L
A286S
N334H
A373T, T373N
R384G
A411T
P423S
M456V
R470K
