# Supplementary material for: Stability-mediated epistasis constrains the evolution of an influenza protein
Source: eLife. 2013 May 14;2:e00631. doi: 10.7554/eLife.00631 (PMC3654441; doi:10.7554/eLife.00631)
Supplement: Figure 8—source code 2. — The code and data are provided in a ZIP file; a README file explains the contents. DOI: http://dx.doi.org/10.7554/eLife.00631.034 [file elife00631s009.zip › elife00631s009/elife00631s009.zip!/Figure_8-source_code_2/FUBAR_results_positive.pdf]

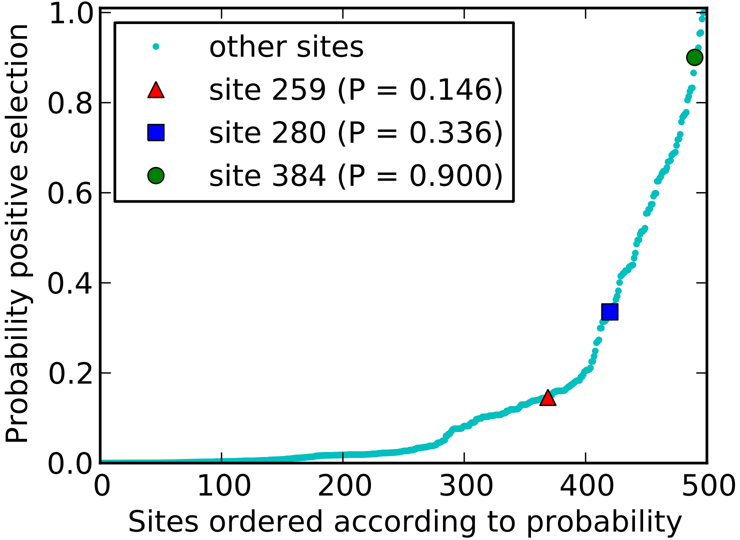

other sites
site 259 (P = 0.146)
site 280 (P = 0.336)
site 384 (P = 0.900)
Probability positive selection
Sites ordered according to probability
0.0
0.2
0.4
0.6
0.8
1.0
0
100
200
300
400
500
